# The Efficacy and Safety of Sintilimab Combined With Nab-Paclitaxel as a Second-Line Treatment for Advanced or Metastatic Gastric Cancer and Gastroesophageal Junction Cancer

**DOI:** 10.3389/fonc.2022.924149

**Published:** 2022-06-01

**Authors:** Jianzheng Wang, Yunduan He, Baiwen Zhang, Huifang Lv, Caiyun Nie, Beibei Chen, Weifeng Xu, Jing Zhao, Xiaojiao Cheng, Qingli Li, Shuiping Tu, Xiaobing Chen

**Affiliations:** ^1^Department of Medical Oncology, The Affiliated Cancer Hospital of Zhengzhou, University and Henan Cancer Hospital, Zhengzhou, China; ^2^Department of Oncology, Renji Hospital, School of Medicine, Shanghai Jiaotong University, Shanghai, China

**Keywords:** gastric cancer, sintilimab, nab-paclitaxel, second-line treatment, immunotherapy

## Abstract

**Background:**

Unresectable advanced or recurrent gastric cancer patients have a poor prognosis. PD-1 monotherapy regimen and PD-1 combined chemotherapy regimen have become the standard third- and first-line treatment for advanced gastric cancer, respectively. However, the status of immune checkpoint inhibitors in the second-line treatment for advanced gastric cancer has not been established. The combination of chemotherapy and anti-PD-1 antibody has been demonstrated to have a synergistic effect. In this study, we aimed to evaluate the efficacy and safety of sintilimab combined with nab-paclitaxel in the second-line treatment for advanced gastric cancer (GC)/gastroesophageal junction (GEJ) cancer patients.

**Patients and Methods:**

We retrospectively analyzed patients with advanced GC/GEJ cancer that progressed after first-line systemic therapies with sintilimab combined with nab-paclitaxel from April 1, 2019 to December 31, 2021. The primary endpoint was progression-free survival (PFS). The secondary endpoints included objective response rate (ORR), disease control rate (DCR), and safety.

**Results:**

Thirty-nine patients were enrolled and eligible for response assessment. Complete response (CR) was not observed, 15 patients achieved partial response (PR), 16 patients had stable disease (SD) and 9 patients had progressive disease (PD). The ORR and DCR were 15 (38.5%) and 31 (79.5%), respectively. Median PFS was 5.4 months (95%CI: 3.072-7.728). PFSs between different subgroups were analyzed. The results showed that gender, age, Human epidermal growth factor receptors 2 (HER2) status, PD-L1 expression, primary tumor site and chemotherapy cycles had no significant effect on PFS. Most of the adverse events (AEs) were of grade 1-2 and manageable. The common treatment-related adverse events of grade 3 or 4 included anemia (12.8%), neutropenia (12.8%), leukopenia (10.3%), hand-foot syndrome (7.7%), thrombocytopenia (7.7%). The potential immune-related adverse events (irAEs) were grade 1 pneumonia (1 pts [2.6%]) and grade 4 hepatitis (1 pts [2.6%]). There were no treatment-related deaths.

**Conclusion:**

These results indicate that sintilimab combined with nab-paclitaxel exhibits good anti-tumor activity and an acceptable safety profile as a second-line treatment for advanced or metastatic gastric cancer. These results warrant further investigation and evaluation to identify patients who can benefit more from the combined treatment strategy.

## Introduction

Advanced gastric cancer has a high mortality rate, and the median survival for metastatic disease is less than 1 year (1–3). Cytotoxic chemotherapy remains the backbone of treatment for advanced gastric cancer (4). Although there are many studies on targeted drugs for gastric cancer, only anti-HER2 drugs such as trastuzumab and anti-angiogenic pathway drugs such as apatinib are currently used in clinical practice (5, 6). Patients with advanced gastric cancer still lack other effective targeted drugs at the molecular level. Immune checkpoint inhibitors have made a breakthrough in the treatment of advanced gastric cancer. Based on the results of prospective clinical studies of ATTRACTION-02 and KEYNOTE-059, immune checkpoint inhibitors have been approved for the third-line treatment of gastric cancer (7, 8). For the first-line treatment of HER2-negative advanced gastric cancer, the Checkmate649 study confirmed that nivolumab combination chemotherapy can bring significant survival benefits to patients and has become a standard treatment recommended by the National Comprehensive Cancer Network (NCCN) Guidelines version 2.2022 and the Chinese Society of Clinical Oncology (CSCO) guidelines 2021 (9). For HER2-positive gastric cancer, the KEYNOTE-811 study showed that pembrolizumab in combination with trastuzumab and chemotherapy could improve the objective response rate (ORR) to 74.4% in patients with HER-2-positive advanced gastric cancer. Therefore, the U.S. Food and Drug Administration (FDA) has accelerated the approval of pembrolizumab combined with trastuzumab and chemotherapy for the first-line treatment of HER-2-positive advanced gastric cancer, and the regimen is also included in the NCCN guidelines version 2.2022 for gastric cancer (10).

However, the second-line treatment of advanced gastric cancer faces many challenges. The status of anti-angiogenic drugs and immunotherapy in the second-line treatment of gastric cancer has not been fully established (11). Currently, the primary second-line treatment for advanced GC/GEJ cancer is chemotherapy such as paclitaxel, docetaxel, and irinotecan monotherapy, or the combination of two-drug chemotherapy regimens based on the drug selection used in first-line treatment (12, 13). However, the overall therapeutic effect is poor. CSCO guidelines only recommend taxanes and irinotecan for second-line treatment, and immunotherapy is only used for patients with microsatellite instability (MSI-H) or deficient mismatch repair (dMMR) (14). However, only a small number of patients with advanced gastric cancer are MSI-H type (15). Therefore, exploring new low-toxic and efficient second-line treatments is a matter of immediate importance.

Sintilimab is a recombinant humanized immunoglobulin G(IgG4) monoclonal antibody against Programmed cell death protein 1 (PD-1). By binding to PD-1 and blocking the binding of PD-1 to PD ligand1 (PD-L1) and PD-L2, it relieves the immunosuppressive effect of PD-1, activates the function of T cells, enhances the immune surveillance and killing ability of T cells against tumors, and generates tumor immune response. Sintilimab is a domestic PD-1 antibody in China and has received approval for the treatment of relapsed or refractory classical Hodgkin lymphoma. Many studies have shown that this drug exhibits favorable anti-tumor effects on a variety of cancer types such as esophageal cancer, gastric cancer, liver cancer, and lung cancer (16–21). Paclitaxel has become very popular due to its broad anticancer activity and is currently used to treat a variety of tumors such as lung, breast and ovarian cancers (22–24). However, due to the poor water solubility of paclitaxel, polyoxyethylene castor oil and ethanol are currently used as solvents, but this solvent system can cause serious side effects such as allergic reactions and altered pharmacokinetic characteristics of paclitaxel. Therefore, researchers have been formulating and developing advanced drug delivery systems for poorly water-soluble anticancer drugs (25–28). Nano drug delivery systems enhance the solubility of paclitaxel are less toxic. Nab-paclitaxel is a 130 nm particle formulation consisting of albumin nanoparticles and paclitaxel linked by a non-covalent bond. *In vivo*, albumin nanoparticles are recognized by albumin receptor gp60; nab-paclitaxel then penetrates vascular endothelial cells by vesicular transport, enters tumor tissue, and binds to secreted acidic and cysteine-rich protein (SPARC), eventually being retained in tumor cells (29–31). One study found that nab-paclitaxel has a modulating effect on cancer- immune cycle, thus providing a potential avenue to enhance the efficacy of immunotherapy as a principle component of combination therapy (32). The strategy of combining immunotherapy with chemotherapy is to kill cancer cells by chemotherapy and change “cold tumors” into “hot tumors” in order to improve the effect of immunotherapy. The chemo-immunotherapy strategy has achieved positive results in the treatment of breast cancer and lung cancer (33–35). The ABSOLUTE study has shown that weekly nab-paclitaxel is not inferior to weekly solvent-based paclitaxel in overall survival, and the CSCO guidelines recommend nab-paclitaxel monotherapy as a second-line treatment for gastric cancer (36). However, the efficacy and safety of sintilimab combined with nab-paclitaxel in the second-line treatment of advanced gastric cancer has not been reported. In this study, we retrospectively collected and analyzed the efficacy and safety of using a PD-1 inhibitor with nab-paclitaxel in patients with advanced gastric cancer, hoping to provide substantive evidence for the a second-line treatment of advanced gastric cancer.

## Patients and Methods

### Patient Enrolment and Eligibility Criteria

This was an observational, single-center, retrospective study. A total 39 patients with GC/GEJ cancer who received sintilimab in combination with nab-paclitaxel as the second-line therapy were enrolled. The patients were recruited from Henan Cancer Hospital from April 1, 2019 to December 31, 2021.

### Therapeutic Regimen and Efficacy Evaluation

Patients received nab-paclitaxel (125mg/m2, d1 & d8) and sintilimab (200mg, d1, ≤ 2 years) every 3 weeks until unacceptable toxicity or disease progression occurs. The combination of sintilimab and nab-paclitaxel was given for at least two cycles, and maintenance treatment was performed after the disease was controlled. Maintenance therapy options were sintilimab monotherapy; sintilimab combined with oral chemotherapy drugs; or sintilimab combined with anti-angiogenesis targeted drugs. Tumor imaging evaluation was performed every two or three cycles according to Response Evaluation Criteria in Solid Tumors (RECIST) version 1.1. All patients signed informed consent forms before treatment.

### Evaluation of Efficacy and Adverse Events

The primary outcome was progression-free survival (PFS), defined as the time from administration of sintilimab combined with nab-paclitaxel until the date of documented disease progression or death from any cause. Secondary outcomes were ORR, which refers to the proportion of patients with at least one confirmed complete response (CR) or partial response (PR) after combined therapy; DCR, defined as the proportion of patients with at least one confirmed CR, PR, or stable disease (SD) after combined therapy; and safety of combined therapy. Other efficacy endpoints included toxicities assessed by the National Cancer Institute Common Terminology Criteria for Adverse Events (NCI CTCAE), version 5.0.

### Statistical Analysis

Statistical analyses were conducted using the software Graphpad prism 8 and SPSS statistics 26. Continuous variables were summarized using medians and ranges, and categorical variables were described using frequency and percentage. PFS was analyzed using the Kaplan-Meier method. PFS between different subgroups was compared using the log-rank test (two-sided). P<0.05 was considered to be statistically significant.

## Results

### Patient Characteristics

Thirty-nine eligible patients with pathologically confirmed advanced GC and GEJ cancer, progression after first-line treatment of fluoropyrimidine and platinum, or progression within six months of neoadjuvant/adjuvant treatment were included in our study. The patient characteristics are summarized in **Table 1**. The median age was 64 years (range 41–78). 53.8% of the patients were>60 years and 66.7% of the patients were male. The pathological types of patients included adenocarcinoma and squamous cell carcinoma, accounting for 38 (97.4%) and 1 (2.6%), respectively. 27 (69.2%) patients had advanced gastric cancer, and the other 12 (30.8%) patients had GEJ adenocarcinoma. All patients were diagnosed as advanced or recurrent. The main metastatic sites included abdominal lymph nodes 8 (20.5%), liver 10 (25.6%), peritoneum 17 (43.6%) and other sites 4 (10.3%). The HER2 status, PD-L1 expression, MMR status, and EBV status of the patients were analyzed. 10 (25.6%) patients were positive for HER2 status; 28 (71.8%) patients were negative; and 1 (2.6%) was unknown. 13 (33.3%) patients were positive for PD-L1 expression; 14 (35.9%) patients were negative; and 12 (30.8%) patients were unknown. With regards to MMR status: MSS 26 (66.7%); dMMR 2 (5.1%); and Unknown 11 (28.2%). 2(5.1%) patients were positive for EBER status,18 (46.2%) patients were negative and 19 (48.7%) patients were unknown. Before 2020, PD-L1, MMR and EBER were not routine detection standards in the pathology department of our hospital due to the limitation of detection reagents and the high price of PD-1 antibodies. Therefore, the status of these three biomarkers in a large number of patients were unknown. The number of cycles of chemotherapy combined with sintilimab ranged from 2 to 7, with 4 and 6 cycles accounting for 13 (33.3%) and 9 (23.1%), respectively. After the combination therapy, 35 (89.7%) patients chose a watch & wait strategy, and the rest of the patients received PD-1-based maintenance therapy: sintilimab monotherapy 2 (5.1%); sintilimab+apatinib 1 (2.6%); sintilimab+xeloda 1 (2.6%).

**Table 1:** Demographic and clinicopathologic characteristics of patients.

Characteristic	N=39
Median age of patients, years (range)	64 (41-78)
≤60, n (%)	18 (46.2%)
>60, n (%)	21(53.8%)
Gender,n (%)	
Male	26 (66.7%)
Female	13 (33.3%)
ECOG performance status, n (%)	
0-1	35 (89.7%)
2	4 (10.3%)
Pathological Type	
Squamous carcinoma	1 (2.6%)
Adenocarcinoma	38 (97.4%)
HER2 status, n (%)	
Positive	10 (25.6%)
Negative	28 (71.8%)
Unknown	1 (2.6%)
PD-L1 status, n (%)	
Positive	13 (33.3%)
Negative	14 (35.9%)
Unknown	12 (30.8%)
MMR, n (%)	
MSS	26 (66.7%)
dMMR	2 (5.1%)
Unknown	11 (28.2%)
EBER, n (%)	
Positive	2 (5.1%)
Negative	18 (46.2%)
Unknown	19 (48.7%)
Primary tumor site, n (%)	
Gastric	27 (69.2%)
GEJ	12 (30.8%)
Metastatic site, n (%)	
Lymph node	8 (20.5%)
Liver	10 (25.6%)
Peritoneum	17 (43.6%)
Others	4 (10.3%)
Cycles of Nab-pactilitaxel combined with PD-1, n (%)	
2	5 (5.1%)
3	7 (17.9%)
4	13 (33.3%)
5	5 (12.8%)
6	9 (23.1%)
7	1 (2.6%)
Maintenance regimen, n (%)	
PD-1	2 (5.1%)
PD-1+Apatinib	1 (2.6%)
PD-1+Xeloda	1 (2.6%)
Watch&Wait	35 (89.7%)

ECOG, Eastern Cooperative Oncology Group performance status; GEJ, Gastroesophageal Junction Tumors; MMR, Mismatch Repair; dMMR, Deficient Mismatch Repair, MSS, Microsatellite Stable. PD-L1 status: positive: CPS≥1, negative: CPS<1.CPS: Combined Positive Score. EBER, Epstein-Barr encoding region.

### Efficacy

**Figure 1:**
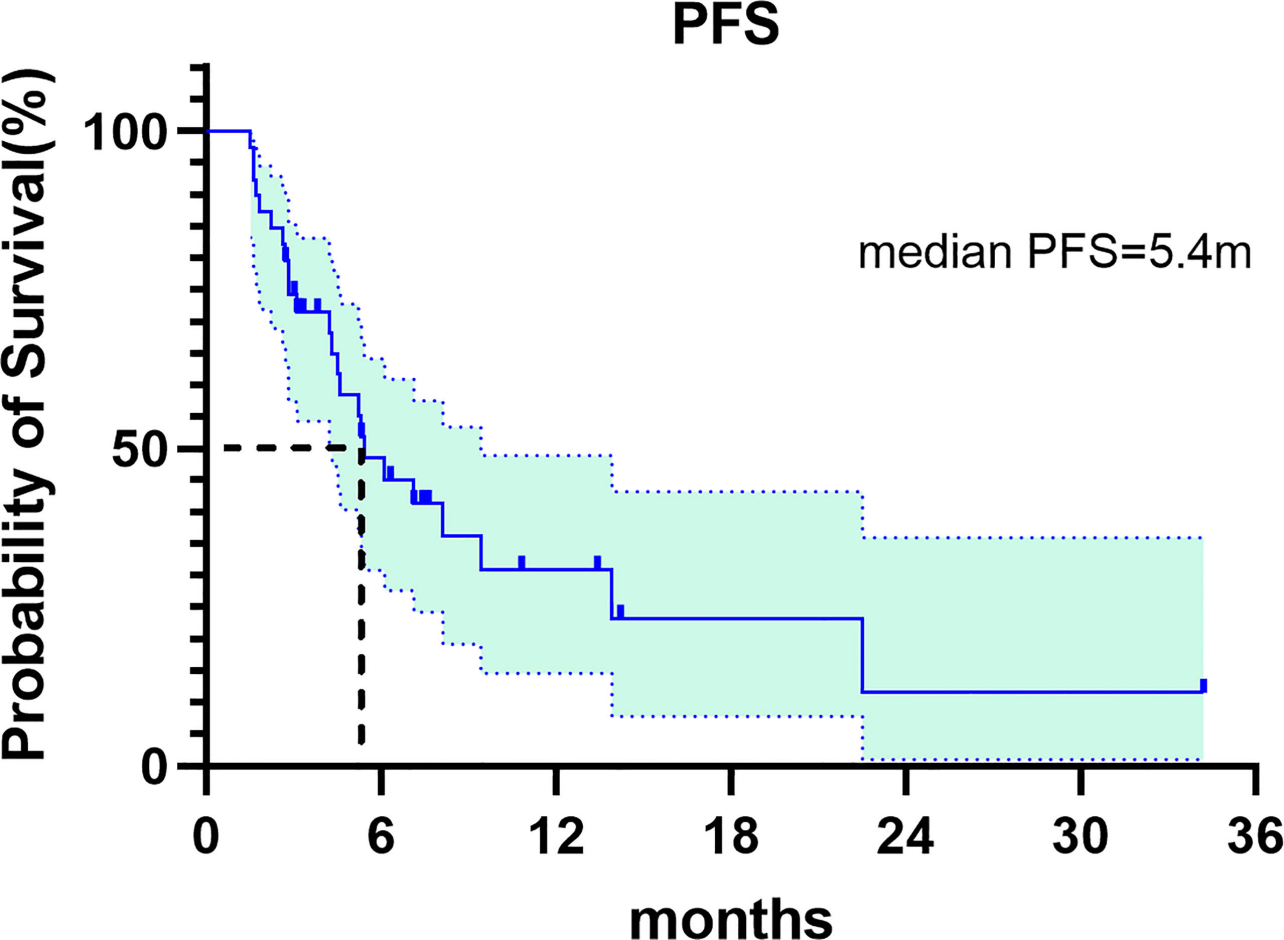
Kaplan-Meier curve of PFS for the overall study population.

**Figure 2:**
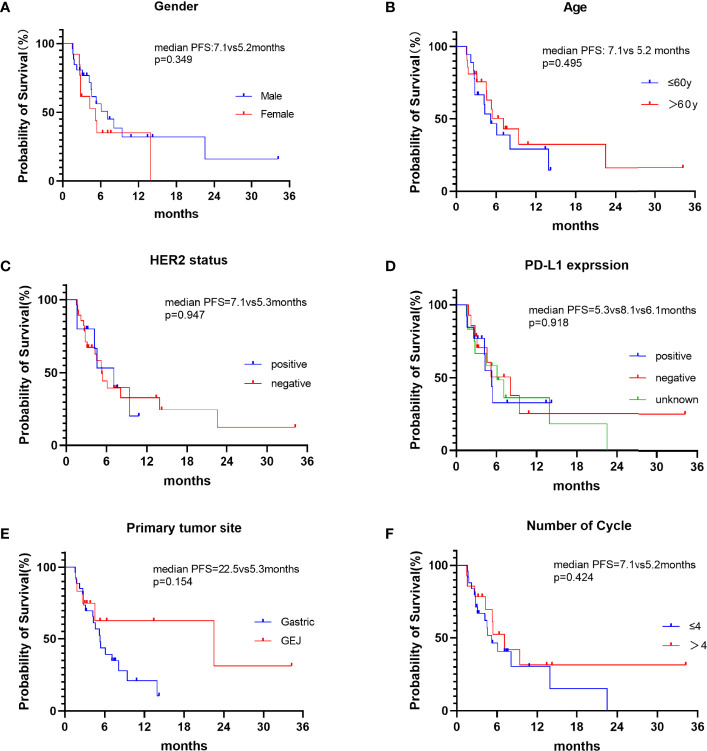
Kaplan-Meier curves of PFS in different subgroups of patients. **(A)** PFS in patients of different gender. **(B)** PFS of patients ≤60y and >60y. **(C)** PFS of HER2-positive and HER2-negative patients. **(D)** PFS of different PD-L1 expression status. **(E)** PFS of different primary tumor sites. **(F)** PFS of different chemoimmunotherapy cycles:≤4 cycles and >4 cycles.

In this study, CR was not observed, 15 patients achieved PR, 16 patients had SD and 9 patients had PD. The ORR and DCR were 15 (38.5%) and 31 (79.5%), respectively (**Table 2**). Median progression-free survival was 5.4 months (95%CI: 3.072-7.728) (**Figure 1**). We further analyzed the relationship between clinicopathological features and prognosis to identify the population that can benefit from combined treatment. Firstly, the PFS of males and females was 7.1 months (95%CI:3.649-10.551) and 5.2 months (95%CI: 1.385-9.015), respectively (p=0.349).The PFS of patients ≤60 years and >60 years were 5.2 months (95%CI:3.012-7.388) and 7.1 months (95%CI:3.878-10.322), respectively (p=0.495), without statistical significance. HER2 status plays an important role in the selection of first-line chemotherapy regimens for gastric cancer (10). Therefore, we further explored the effect of HER2 status on the prognosis of second-line chemoimmunotherapy. The PFS of HER2-positive and HER2-negative patients was 7.1months (95%CI: 3.367-10.833) and 5.3 months (95%CI:4.018-6.582), respectively. The PFS of HER2-positive patients was longer than that of HER2-negative patients, but the difference was not statistically significant (p=0.947). We explored the effect of PD-L1 expression on PFS. The results showed that the median PFS of PD-L1-positive, PD-L1-negative, and PD-L1-unknown patients was 5.3months (95%CI: 3.744-6.856), 8.1months (95%CI: 3.371-12.829) and 6.1 months (95%CI: 2.649-9.551)(p=0.918), respectively. The results suggest that the efficacy of second-line chemotherapy immunity was not affected by PD-L1 expression. We also observed the effect of the primary tumor site on PFS. The results showed that the PFS of GEJ cancer was longer than that of gastric cancer; 22.5 months (95%CI: 0.000-48.927) and 5.3 months (95%CI: 4.084-6.516) (p=0.154), respectively. However, the difference was not statistically significant. Finally, we analyzed the effect of chemoimmunotherapy cycles on PFS. The results showed that PFS was numerically longer with > 4 cycles than that seen with ≤ 4 cycles, measuring at 7.1 months (95%CI: 4.378-9.822) and 5.2 months (95%CI: 3.091-7.309) (p=0.424), respectively (**Figure 2**).

**Table 2:** Tumor Response.

Best Response	N = 39, n (%)
CR	0 (0%)
PR	15 (38.5%)
SD	16 (41.0%)
PD	9 (23.1%)
ORR	15 (38.5%)
DCR	31 (79.5%)

### Safety

In terms of safety, most of the adverse events (AEs) were grade 1-2 (**Table 3**). The most common treatment-related adverse events (TRAEs) of grade 3-4 were anemia 5 (12.8%), neutropenia 5 (12.8%), leukopenia 4 (10.3%), hand-foot syndrome 3 (7.7%), thrombocytopenia 3 (7.7%), asthenia 2 (5.2%), increased aspartate aminotransferase 1 (2.6%), and increased alanine aminotransferase 1 (2.6%). No treatment-related deaths occurred. The potential immune-related adverse events (irAEs) were grade 1 pneumonia and one patient (2.6%) developed this. Only one patient with liver metastasis developed grade 4 hepatitis (2.6%).

**Table 3:** Treatment-Related Adverse Events.

AE	All Grade, n (%)	Grade≥ 3, n (%)
Anemia	24 (61.5%)	5 (12.8%)
Leukopenia	23 (59.0%)	4 (10.3%)
Neutropenia	15 (38.5%)	5 (12.8%)
Hand-foot syndrome	10 (25.6%)	3 (7.7%)
AST increased	9 (23.1%)	1 (2.6%)
ALT increased	9 (23.1%)	1 (2.6%)
Thrombocytopenia	9 (23.1%)	3 (7.7%)
Asthenia	6 (15.4%)	2 (5.2%)
Nausea	4 (10.3%)	0
Vomiting	3 (7.7%)	0
Mucositis	2 (5.2%)	0
Proteinuria	1 (2.6%)	0
irAE		
-Pneumonia	1 (2.6%)	0
-Hepatitis	0	1 (2.6%)

AST, Aspartate aminotransferase; ALT, Alanine aminotransferase; irAEs, immune-related adverse events.

## Discussion

At present, there are standard regimens for the first- and third-line immunotherapy for advanced gastric cancer, but the second-line immunotherapy for gastric cancer is still being investigated (7, 8, 37, 38). Phase III KEYNOTE-061 study of second-line immunotherapy has not yielded positive results. The results showed that second-line treatment with pembrolizumab monotherapy failed to significantly improve overall survival in patients with PD-L1 CPS ≥1 compared with standard chemotherapy paclitaxel (11). Based on the data from the KEYNOTE-158 trial and subsequent retrospective analysis, pembrolizumab can be used for second-line or subsequent treatment of MSI-H/dMMR or TMB high (≥10mutations/megabase) gastroesophageal tumor and is recommended in the NCCN guidelines (14, 39–41). However, it should be noted that patients with gastroesophageal cancer were not included in the KEYNOTE-158 trial. Also, MSI-H/dMMR is uncommon in patients with metastatic gastric cancer (15, 42), therefore, there is an urgent need to explore effective immunotherapy regimens suitable for the general patient-wide population. This is the first retrospective study to assess the efficacy and safety of immunotherapy (sintilimab) plus single-agent chemotherapy (nab-paclitaxel) combination regimen as the second-line treatment for GC/GEJ cancer. Our findings provide new data to support the second-line immunotherapy for gastric cancer and represent a highly innovative approach. The ORR and DCR of sintilimab plus nab-paclitaxel combination therapy for GC/GEJ cancer were 38.5% and 79.5% respectively. The median PFS was 5.4 months. The toxicity was tolerable, and most patients could continue their treatment until disease progression.

Currently, there is very little data on prospective clinical studies of second-line immunotherapy for gastric cancer. Yan Song et al. reported the efficacy and safety of HX008 (PD-1 antibody) combined with irinotecan in second-line immunotherapy combined with chemotherapy for gastric cancer. This is the first prospective clinical study on PD-1 monoclonal antibody combined with single-agent chemotherapy. The ORR was 27.6% (95% CI 16.1%-39.1%); and the DCR was 60.3% (95%CI 46.4%-73.0%). The ORRs of PD-L1-positive (CP ≥ 1) and -negative (CPS < 1) tumor patients were 38.5% and 37.5%, respectively. The median PFS was 4.2 months (95%CI 2.2-5.5) (43, 44). The phase III clinical study of HX008 in combination with irinotecan versus placebo in combination with irinotecan for the second-line treatment of advanced GC/GEJ cancer is currently in the recruitment phase. Anti-PD-1 monoclonal antibody combined with anti-angiogenesis therapy has anti-tumor activity and works synergistically. Chao jing et al. explored the efficacy and safety of the combination therapy of camrelizumab, apatinib, and S-1 in patients with GC or GEJ adenocarcinoma. The results showed that the ORR was 29.2% (95%CI 14.9–49.2%). The median PFS was 6.5 months (95%CI 6.01–6.99) (45). Nakajima TE et al. reported the results of a multicenter phase I/II study of nivolumab, paclitaxel combined with ramucirumab as a second-line treatment regimen for patients with advanced gastric cancer. The ORR was 37.2% (95%CI, 23.0–53.5%), the median PFS was 5.1 months (95%CI, 4.5–6.5 months), and the median OS was 13.1 months (95%CI, 8.0–16.6 months) (46). In ASCO-GI 2022, Zhichao Jiang et al. reported on the preliminary findings of the prospective phase II clinical study of sintilimab combined with nab-paclitaxel in the second-line treatment of gastric cancer. The ORR was 41.9%, and DCR was 83.9%. The median PFS was 5.2 months (95%CI 3.556-6.848) (47). Our findings are consistent with those of ZhiChao Jiang et al. In general, in these phase II clinical studies, the PFS of three-drug and two-drug combination regimens is within a similar range. The treatment regimen needs to be determined after serious consideration given to factors such as the pharmaco-economics, toxicity, and patient tolerance. Perhaps “less is more” is a good strategy.

**Figure 3:**
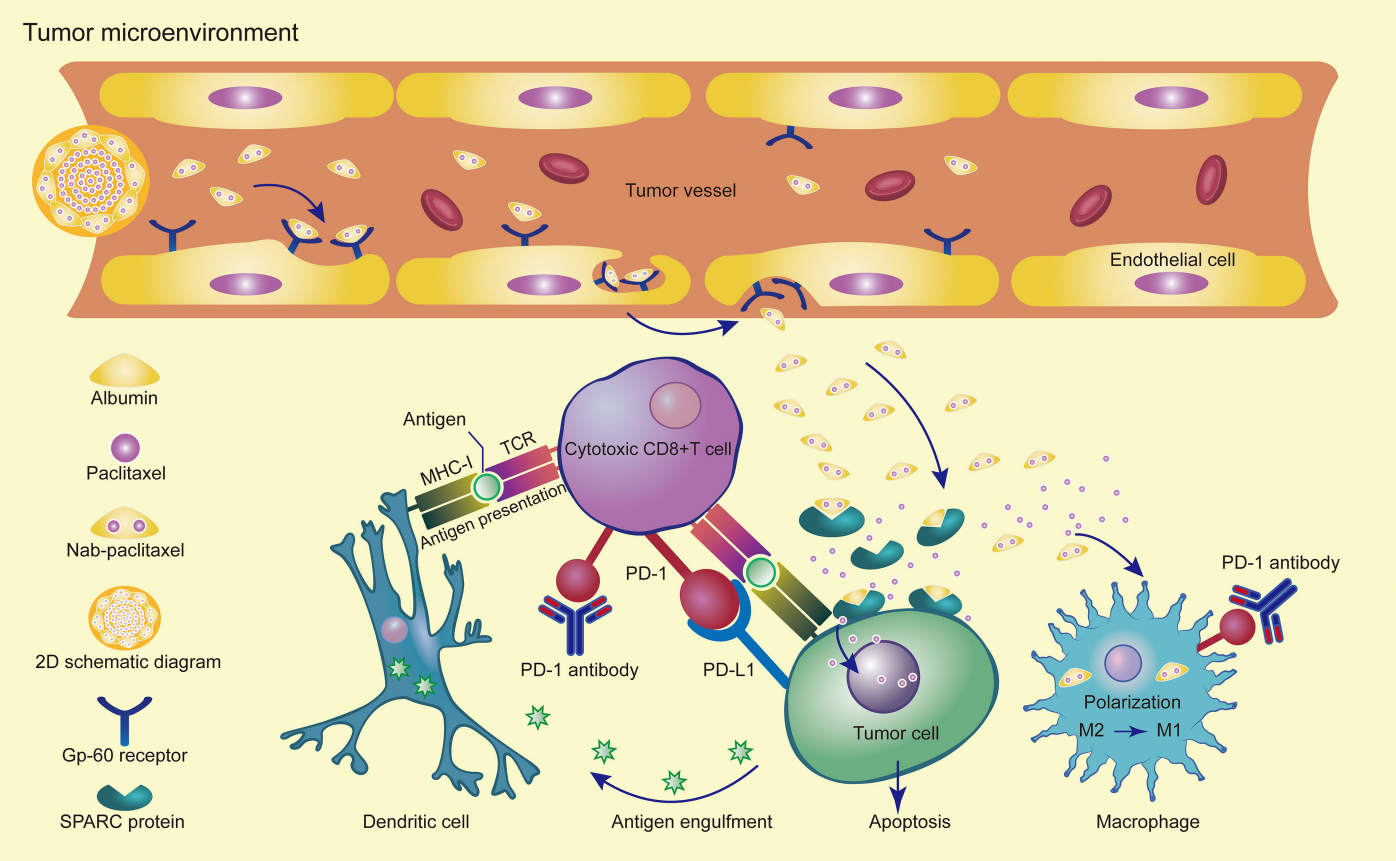
Synergistic effects of PD-1 antibody in combination with nab-paclitaxel in the tumor microenvironment.

The second-line treatment of advanced gastric cancer has many challenges and several elements need to be taken into consideration, but the patient’s physical condition is the main focus of our attention. In many cases, patients’ physical status decreases after the first-line treatment, and only 32% of patients can enter second-line treatment. As such, early clinical trials were designed to compare single-agent chemotherapy with best supportive care (BSC) in second-line treatment (48–50). Both NCCN and CSCO guidelines use chemotherapy alone as a preferred treatment for both Her2-positive and Her2-negative patients. From the perspective of patient tolerance, single-agent chemotherapy combined with immunotherapy is a good strategy for patients entering second-line treatment. Checkpoint inhibitors were added to standard-of-care chemotherapy and have achieved success in multiple clinical trials. Potential mechanisms for the synergistic effect of combined immunotherapy and chemotherapy include immunogenic tumor cell death, anti-angiogenesis, selective depletion of myeloid immunosuppressive cells, reduction in regulatory T cells, and promotion of effective T cell proliferation (51, 52). Nab-paclitaxel can regulate the cancer-immune cycle by enhancing antigen presenting cells (APCs) antigen presentation ability, indirectly promoting T cell activation, thereby reversing tumor microenvironment immunosuppression, and cooperating with cytotoxic T lymphocytes (CTLs) to kill tumor cells. The multi-faceted induction of nab-paclitaxel in the cancer-immune cycle may provide a new approach to tumor treatment (32). Studies have found that nab-paclitaxel internalization by tumor-associated macrophages can promote the activation of M1 macrophages and increase the MHC II+CD80+CD86+M1 macrophage (53). PD-1 expression by tumor-associated macrophages can inhibit phagocytosis and tumor immunity (54). PD-1 expression in T cells and TAMs inhibits anti-tumor immunity. PD-1 antibody induces innate immunity through TAM phagocytosis and adaptive immunity through T cell cleavage activity (55) (**Figure 3**). We conducted a retrospective analysis of phase III clinical studies of taxane-containing second-line treatment of gastric cancer. The WJOG 4007 trial compared the efficacy of weekly paclitaxel with biweekly irinotecan in patients with refractory advanced gastric cancer treated with fluoropyrimidine combined with platinum. The results showed that the ORR was 20.9% in the paclitaxel group and 13.6% in the irinotecan group (P =0.24). The median PFS was 3.6 months in the paclitaxel group and 2.3 months in the irinotecan group (P =0.33) (13). GATSBY study aims to evaluate the efficacy of trastuzumab emtansine (TDM-1) in patients previously treated for HER2-positive advanced gastric cancer.The ORR was 20.6% in TDM-1 group and 19.6% in taxane group.The median PFS was 2.7 months in TDM-1 group and 2.9 months in taxane group (56).RAINBOW study showed that the ORR was 28% in ramucirumab plus paclitaxel cohort and 16% in placebo plus paclitaxel cohort.The PFS was 4.4 months vs 2.86 months,respectively (57). The RAINBOW-Asia study is a bridge study of the RAINBOW study and the results showed consistent efficacy and safety with the RAINBOW study. The PFS was 4.14 months in ramucirumab plus paclitaxel group and 3.15 months in placebo plus paclitaxel group. From these studies, we can see that the objective response rate of single-agent paclitaxel is less than 30%, and the median PFS is less than 5 months, which is inferior to the results of our study and those of Zhichao Jiang et al. Therefore, it is suggested that there is a synergistic effect between nab-paclitaxel and sintilimab. Solvent-based paclitaxel requires advanced administration of antihistamines and steroid hormones with strict dosing and frequency (58). However, nab-paclitaxel overcomes the disadvantage that hormone pre-treatment may weaken the efficacy of immunotherapy, and can rapidly enrich in cancer tissues. It naturally becomes the first choice for combination with immunotherapy.

AEs associated with sintilimab and nab-paclitaxel were in general of mild severity and manageable. The main AEs were anemia and leukopenia caused possibly by nab-paclitaxel. Myelosuppression is managed easily by medical oncologists. The most common immune-related AEs were rash, fatigue, pruritus, hypothyroidism, proteinuria, and decreased appetite (59). In our study, these irAEs were not observed. Only one patient presented with grade1 immunotherapy-associated pneumonia and one patient with liver metastasis developed grade 4 hepatitis.

This research has some limitations, that included but was not limited to the relatively small number of patients, short follow-up time, failure to collect the overall survival data of patients, and the lack of further investigation into the impact of MMR status and EBER status on survival.

Additionally, our study identified some areas that need further exploration. Firstly, the potential synergistic mechanism between these two drugs needs to be further elucidated. It is unclear as to the optimum number of cycles that would constitute the best regimen for nab-paclitaxel (60). Second, it is unclear which drugs play a major role in this combination. Finally, given the encouraging effect of this combined treatment, a clinical study with a larger sample size should be carried out to determine which patients will benefit the most from this treatment.

## Conclusion

Our results showed that the application of sintilimab combined with nab-paclitaxel as the second-line treatment for advanced or metastatic GC/GEJ cancer patients is effective and safe. We look forward to more stage III clinical studies to further confirm our findings.

## Data Availability Statement

The original contributions presented in the study are included in the article/supplementary material. Further inquiries can be directed to the corresponding authors.

## Author Contributions

JW and XBC treated the patients. JW, XJC, HL, CN, BC, WX, JZ, and YH collected the data. JW wrote the original draft. BZ, QL, and ST analyzed the data and revised the draft. All authors contributed to the article and approved the submitted version.

## Funding

This work was supported by the Science and Technique Foundation of Henan Province (No. 202102310121 for JW), the Science and Technique Foundation of Henan Province (No. 222102310424 for BC), the Medical Science and Technology Co-construction Project of Henan Province (No. LHGJ20200167), the 1000 Talents Program of Central plains (No. 204200510023 for XBC), and the Sate Key Laboratory of Esophageal Cancer Prevention & Treatment (No. Z2020000X for XBC).

## Conflict of Interest

The authors declare that the research was conducted in the absence of any commercial or financial relationships that could be construed as a potential conflict of interest.

The reviewer FR declared a shared parent affiliation with the authors BZ, XJC, QL, ST, and XBC to the handling editor at the time of review.

## Publisher’s Note

All claims expressed in this article are solely those of the authors and do not necessarily represent those of their affiliated organizations, or those of the publisher, the editors and the reviewers. Any product that may be evaluated in this article, or claim that may be made by its manufacturer, is not guaranteed or endorsed by the publisher.
